# Cases of Acute Flaccid Paralysis Associated with Coxsackievirus A2: Findings of a 20-Year Surveillance in the Russian Federation

**DOI:** 10.3390/microorganisms10010112

**Published:** 2022-01-06

**Authors:** Olga E. Ivanova, Armen K. Shakaryan, Nadezhda S. Morozova, Yulia A. Vakulenko, Tatyana P. Eremeeva, Liubov I. Kozlovskaya, Olga Y. Baykova, Elena Y. Shustova, Yulia M. Mikhailova, Natalia I. Romanenkova, Nadezhda R. Rozaeva, Natela I. Dzhaparidze, Nadezhda A. Novikova, Vladimir V. Zverev, Lyudmila N. Golitsyna, Alexander N. Lukashev

**Affiliations:** 1 Federal State Autonomous Scientific Institution “Chumakov Federal Center for Research and Development of Immune-and-Biological Products of the Russian Academy of Sciences” (Institute of Poliomyelitis) (FSASI “Chumakov FSC R&D IBP RAS”), 108819 Moscow, Russia; armen2@mail.ru (A.K.S.); poliom_ldms@mail.ru (T.P.E.); lubov_i_k@mail.ru (L.I.K.); baykovaaa@mail.ru (O.Y.B.); shustova_eu@chumakovs.su (E.Y.S.); 2 Department of Organization and Technology of Production of Immunobiological Preparations, Institute for Translational Medicine and Biotechnology, First Moscow State Medical University (Sechenov University), 119991 Moscow, Russia; 3 Pirogov Russian National Research Medical University, 119121 Moscow, Russia; 4 Federal Budget Institution of Healthcare of Rospotrebnadzor “Center for Hygiene and Epidemiology in Moscow”, 129626 Moscow, Russia; oki@fcgie.ru (N.S.M.); epid20@bk.ru (Y.M.M.); 5 Martsinovsky Institute of Meidcal Parasitology, Tropical and Vector-Borne Diseases, First Moscow State Medical University (Sechenov University), 119991 Moscow, Russia; vjulia94@gmail.com; 6 Saint-Petersburg Pasteur Institute, 197101 Saint-Petersburg, Russia; romanenkova@pasteurorg.ru (N.I.R.); romanenkova@pasteurorg.r (N.R.R.); 7 Federal Budgetary Institution of Healthcare of Rospotrebnadzor “Center for Hygiene and Epidemiology in the Vladimir Region”, 600005 Vladimir, Russia; nid0209@icloud.com; 8 Academician I.N. Blokhina Nizhny Novgorod Scientific Research Institute of Epidemiology and Microbiology, 603950 Nizhny Novgorod, Russia; mevirfc@rambler.ru (N.A.N.); arceo@yandex.ru (V.V.Z.); lyudmila_galitzina@mail.ru (L.N.G.)

**Keywords:** acute flaccid paralysis (AFP), coxsackievirus A2 (CV-A2), poliomyelitis, polioviruses, non-polio enteroviruses, epidemiological surveillance

## Abstract

Surveillance for acute flaccid paralysis syndrome (AFP) in children under 15 is the backbone of the Global Polio Eradication Initiative. Laboratory examination of stool samples from AFP cases allows the detection of, along with polioviruses, a variety of non-polio enteroviruses (NPEV). The etiological significance of these viruses in the occurrence of AFP cases has been definitively established only for enteroviruses A71 and D68. Enterovirus Coxsackie A2 (CVA2) is most often associated with vesicular pharyngitis and hand, foot and mouth disease. Among 7280 AFP cases registered in Russia over 20 years (2001–2020), CVA2 was isolated only from five cases. However, these included three children aged 3 to 4 years, without overt immune deficiency, immunized with 4–5 doses of poliovirus vaccine in accordance with the National Vaccination Schedule. The disease resulted in persistent residual paralysis. Clinical and laboratory data corresponded to poliomyelitis developing during poliovirus infection. These findings are compatible with CVA2 being the cause of AFP. Molecular analysis of CVA2 from these patients and a number of AFP cases in other countries did not reveal association with a specific phylogenetic group, suggesting that virus genetics is unlikely to explain the pathogenic profile. The overall results highlight the value of AFP surveillance not just for polio control but for studies of uncommon AFP agents.

## 1. Introduction

Coxsackievirus A2 (CVA2) belongs to the genus *Enterovirus* of the *Picornaviridae* family and is a representative of the species *Enterovirus A* [1]. CVA2 is widespread throughout the world, causing outbreaks [2,3,4] or sporadic cases of disease [5]. It is most commonly associated with vesicular pharyngitis, hand, foot and mouth disease (HFMD) [4,6] and pleurodynia [7]. Rare events of CVA2 infection in the form of myocarditis [8,9] and a case of fulminant type 1 diabetes mellitus associated with CVA2 [10] have been described. CVA2 has also been reported to cause infectious diseases of the nervous system, such as meningitis, encephalitis, and myelitis [7]. The latter can manifest as acute flaccid paralysis syndrome (AFP), accompanied by persistent movement disorders [7], and is clinically (without laboratory tests) indistinguishable from paralytic poliomyelitis caused by poliovirus [11].

Acute flaccid paralysis (AFP) is a clinical syndrome characterized by rapid onset of weakness of the limbs, progressing to maximum severity within several days to weeks [11]. The term “flaccid” indicates the weakness of muscles and decreased tone and tendon reflexes. AFP can be a manifestation of many diseases of various etiologies—both infectious (viral, bacterial) and non-infectious (neurological lesions, oncological diseases, trauma, exposure to toxins, and a number of others) [11]. More than 50% of AFP cases are due to Guillain-Barrée syndrome—an autoimmune disease of the peripheral nervous system, which can be caused by many factors [11,12]. AFP syndrome is a specific feature of paralytic poliomyelitis, and global AFP surveillance became one of the cornerstones of the Global Polio Eradication Program initiated by the World Health Organization (WHO) in 1988 [13]. Each AFP syndrome case is investigated to establish its etiology and any potential link to polio infection, and poliovirus isolation is attempted for each patient. A small but significant fraction of AFP patients yield non-polio enteroviruses, represented by over 100 types comprising four species.

The proportion of AFP cases from which non-polio enteroviruses (NPEV) were isolated varies from country to country: 4.3% in Tunisia [14], 6.12% in Poland [15], 19.4% in Italy [16], and 20.8% in the Philippines [17]. The etiological role of NPEV is not always obvious, since the WHO-recommended AFP case investigation algorithm consists of examining stool samples [18], and the isolation of ubiquitously present NPEV from a non-sterile specimen may be an accidental finding. The small numbers of AFP cases associated with a particular NPEV type and the presence of the same viruses in healthy humans make it difficult to prove their etiological role. One argument may come from a meta-analysis by Suresh et al. [19], reporting that enterovirus A71 (EV-A71) was most frequently associated with AFP cases, followed by echovirus 13 and echovirus 11. These types are widespread but not the most common [20,21], thus supporting their role as AFP causative agents.

The detection of CVA2 virus from AFP cases is apparently a rare event [14,22,23], but in a study carried out in Brazil, CVA2 was one of the leading types of NPEVs isolated from AFP cases [24]. Among the 7280 AFP cases registered in the Russian Federation over 20 years (2001–2020) [25], CVA2 was isolated from only five cases. Here, we present the epidemiological, virological and clinical characteristics of these AFP cases.

## 2. Materials and Methods

### 2.1. Case Identification

AFP cases have been identified in the framework of the Russian Federation’s national polio and AFP surveillance program, which was initiated in 1996 as a part of the WHO Global Polio Eradication Program [26]. According to routine, AFP cases were registered in the epidemiological database. Two fecal samples (obligatory, at 24–48 h intervals) were taken from each patient. In our investigation, an oropharyngeal swab was additionally taken from one patient.

### 2.2. Epidemiological and Clinical Data

Epidemiological information on the AFP cases was obtained from the cards of the epidemiological investigation. Data on the clinical presentations were obtained from the medical documentation during hospitalization or personal observations.

### 2.3. Virological Investigation

Fecal samples were examined in virological laboratories of the Russian polio surveillance network in accordance with the WHO recommendations [18]. To isolate viruses, we used cell cultures RD, Hep-2 and L20B obtained from sources approved by the WHO. Virus identification was performed using a neutralization assay according to the standard WHO protocol [18] with polyclonal sera (RIVM, Bilthoven, the Netherlands) for the identification of poliovirus type 1–3, pools A–G for the identification of 20 EV-B types and one parechovirus and pools H–R for the identification of 30 non-polio enteroviruses of different types (11 EV-A, 12 EV-B, 5 EV-C, 1 EV-D and one parechovirus). Virus isolates from all five patients were sequenced in the partial VP1 genome region as described previously [27].

### 2.4. Phylogenetic Studies

All available CVA2 sequences (as of 12 January 2021) were extracted from Genbank (*n* = 538). In addition, we used 15 CVA2 sequences identified from viruses collected in Russia as a part of enterovirus monitoring [28] and AFP surveillance [26]. Sequences identified from archive samples for this study were deposited to Genbank with accession numbers JQ518448-JQ518450, JQ518465-JQ518470, JX139774-JX139777 and OL381925-OL381989. The fragment of VP1 gene referred to as the typing fragment routinely used for enterovirus identification [29] was most common in this dataset and was used for further studies. The alignment of the typing fragment was automatically generated using in-house Python scripts [30]. The preparation of alignment included the following steps:

(1) Collection date and place of sequences extracted from the Genbank database were retrieved from Genbank annotation. Sequences with missing metadata were omitted.
(2) Too-short (250 nt) and too-long sequences (over 8000 nt) were removed from the dataset.
(3) Both sequences downloaded from Genbank and 15 sequences of isolates collected in Russia were aligned using MAFFT v.7.304 [31].
(4) Typing VP1 sequence was excised from an alignment automatically according to a reference sequence.
(5) Sequences with multiple ambiguous nucleotides and obvious errors were omitted.

The resulting alignment contained 248 sequences of VP1 typing fragment and was further used for phylogenetic analysis.

A maximum likelihood (ML) phylogenetic tree was reconstructed using IQ-TREE v.1.6.12 [32] with 1000 ultrafast bootstrap replicates (UFBoot) [33]. The best-fit nucleotide substitution model (TIM2e + I + G4) was chosen according to the Bayesian information criterion yielded by ModelFinder [34] implemented in IQ-TREE. To evaluate the temporal signal of the sequence dataset, an ML tree rooted by the midpoint was further analyzed using TempEst (formerly Path-O-Gene) software [35].

Bayesian phylogenetic analysis was performed using BEAST v1.10.4 [36]. The best-fit partitioning scheme (1, 2 + 3) and substitution models (K80 + I + G, SYM + I + G) for Bayesian analysis were chosen according to the Bayesian Information Criterion using the PartitionFinder 2 program [37]. Then, we compared different combinations of coalescent tree priors (coalescent constant size, coalescent exponential growth, Bayesian skygrid) and molecular clock models (strict, relaxed log-normal, relaxed exponential) by the Bayes factor (BF) test. Marginal likelihoods were calculated using the path sampling/stepping stone procedure implemented in BEAST [38]. The combination of the coalescent exponential tree prior and relaxed log-normal molecular clock model was strongly favoured (log BF > 13). Three independent analyses were run for 50 million generations, and trees and parameters were sampled every 5000 generations. Convergence was inspected using Tracer 1.6 [39]. The maximum clade credibility (MCC) tree was annotated with TreeAnnotator v1.8.2 using a burn-in of 5 million generations. The tree was visualized with FigTree v1.4.2 [40].

### 2.5. Ethical Statement

Samples were collected as a part of the Russian state program for polio surveillance. According to the national regulations, the use of anonymous samples and data from state epidemiological surveillance does not require informed consent. Clinical data were extracted from case histories, and no interventions were undertaken specifically for this study.

## 3. Results

### 3.1. Clinical and Epidemiological Characteristics of Cases

The main descriptive characteristics of AFP cases are shown in the Table 1.

AFP cases with CVA2 isolation were detected in five regions of the central part of the Russian Federation in 2008 (two cases), 2015 (one case) and 2019 (two cases) in two girls and three boys. The age of the children ranged from 1 to 4 years (2.6 ± 1.14). All cases developed in autumn—four cases in September and one in October. Before disease onset, children had completed vaccination against poliomyelitis in accordance with the contemporary national schedule of preventive vaccinations of the Russian Federation (except for patient H.A., who received two doses of OPV out of the three required at his age). Between 5 and to 24 months (12.4 ± 7.23) had passed from the last vaccination to AFP onset.

In all children except H.A., the disease began with fever (37.8–39.0 °C). None of the children had skin lesions (hand, foot and mouth disease, HFMD). Motor disorders appeared 3.6 ± 3.78 days after disease onset (interval from 1 to 10 days). The localization of paresis varied: tetraparesis (one case, K.O.), upper paraparesis (one case, N.T.), lower paraparesis (one case, H.A.) and lower monoparesis (two cases, K.G., D.A.). In all cases, the paralysis was asymmetric, being more pronounced in the proximal and peripheral parts, with decreased muscle tone and absence of tendon reflexes. There were no signs of sensitive disorders. In one case (K.G), a short-term episode of encopresis and enuresis occurred. Cerebrospinal fluid (CSF) was examined in three cases. Increased cell level with normal albumin level on the first examination of CSF was replaced with albuminocytological dissociation over 7 days (increased protein concentration with normal cell count) in patient K.O. Albuminocytological dissociation was also detected in K.G during the study in the acute period. In N.T., no pathological CSF changes were found on day 90. During the 60-day follow-up, stable motor disorders in the affected limbs persisted in three cases (N.T., D.A., and K.O.). An MRI (magnetic resonance imaging) examination of the spinal cord was performed in two patients (K.O. and N.T.). No pathological changes were found in either case on days 7 and 20 after the paralysis onset, respectively. Re-examination on day 90 in patient N.T. detected focal changes in the cervical part of the spinal cord. Patients D.A., N.T. and K.O. had an electromyographic study with signs of damage of the anterior horns of the spinal cord. Thus, in at least three cases (D.A., N.T. and K.O.), we observed an acute onset with fever; the development of acute flaccid asymmetry, predominantly proximal paresis of the extremities, with an increase period of less than 4 days; residual paralysis after 60 days and changes in electromyography corresponding to damage to the anterior horns of the spinal cord. A CSF study of patients N.T. and K.O revealed inflammatory changes. These clinical, laboratory and instrumental data meet the diagnostic criteria for acute infection-related lesions of the anterior horns of the spinal cord, very similar to the clinical characteristics of poliovirus infection. According to the currently accepted regulations [41,42], such patients may be considered as cases of paralytic poliomyelitis.

Clinical features of these three patients were also poorly compatible with other causes of AFP. The most common cause of AFP is Guillain-Barré syndrome (GBS)—a condition that develops 2–4 weeks after a nonspecific infection or an infection caused by *Campylobacter jejuni*, against a background of normal temperature, accompanied by the symmetrical, predominantly distal paresis of all extremities, with sensory disorders. The duration of the increase in paresis in GBS is usually 7–14 days, with albuminocitologycal dissociation in the CSF, and EMG signs of neural involvement [11].

In patients D.A., N.T. and K.O., AFP coincided with fever, pareses were asymmetric, mostly proximal, without sensory disturbances, abd the motor dysfunction increase period was less than 4 days with residual movement disorders that persisted after a 60 day follow-up. Electromyography in patients D.A., N.T. and K.O. revealed signs of damage of the anterior horns of the spinal cord. Thus, the clinical features in patients N.T., D.A. and K.O. were clinically and instrumentally not compatible with GBS, traumatic neuritis, ischemic damage of nerves and other neurological disorders.

The disease of patient H.A. was characterized by a febrile onset with a period of increase in symmetrical flaccid paralysis of the lower extremities for 10 days, without residual effects at 60 day follow-up. The disease of patient K.G. proceeded with the clinical characteristics of transverse myelitis (a combination of fever with acute flaccid paralysis of the extremities, dysfunction of the pelvic organs, albuminocytological dissociation in the CSF at the onset), but this was not accompanied by the formation of the persistent residual paralysis that is one of the key features of poliomyelitis. Although CVA2 was isolated from the latter two cases, the clinical features were not compatible with paralytic poliomyelitis.

### 3.2. Virological Investigation

CVA2 viruses were isolated in RD cell culture from fecal samples of all children. For patient N.T., the virus was also isolated from an oropharyngeal swab. The patients were tested negative for other pathogens potentially causing the AFP syndrome: tick-borne borreliosis (Lyme disease), herpesvirus infection and tick-borne encephalitis.

### 3.3. Phylogenetic Analysis

**Figure 1:**
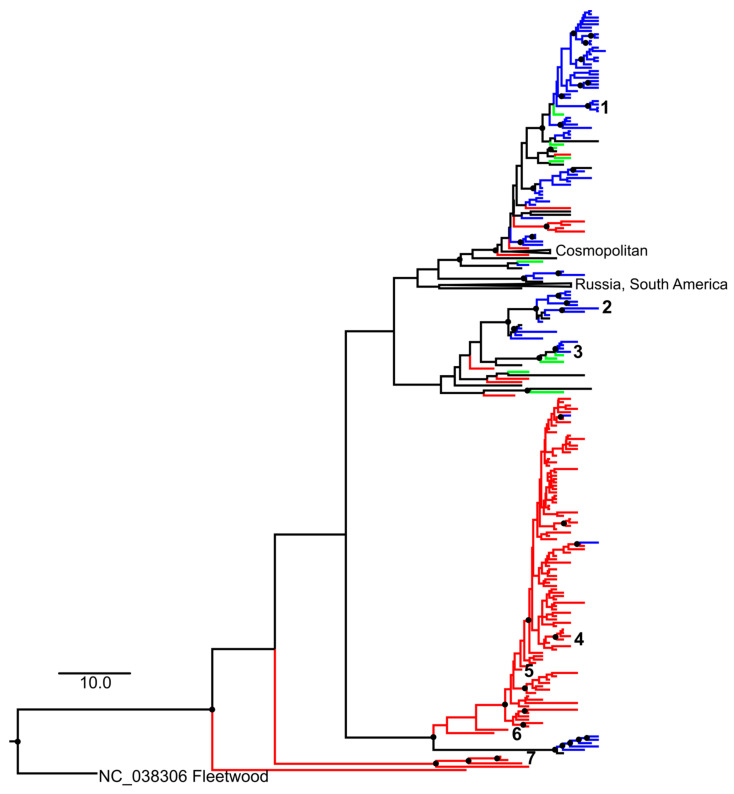
MCC tree for CVA2 VP1 gene typing fragment sequences available in GenBank. The scale bar corresponds to 10 years. Nodes with posterior probabilities above 0.95 are indicated as filled circles. Branches leading to sequences collected in Russia, East Asia (China, Japan) and the European Union are colored in blue, red and green, respectively. Isolates associated with AFP are numbered as follows: (1)—patient N.T.; (2)—patient K.O.; (3)—patient D.A.; (4)—2014, Taiwan, sequence described earlier [43]; (5)—2008, China, no reference; (6), (7)—China, 2006, no reference. Scale bar indicates time in years. Groups that did not contain AFP-associated CVA2 were collapsed.

The global distribution of CVA2 genetic variants was, in general, associated with geographic location (Figure 1). There was a large and distinct group of Chinese CVA2 isolates, and relatively few transfers between Europe and Asia. CVA2 isolates associated with AFP in this and other studies were scattered among other CVA2 sequences on the phylogenetic tree. There was no association of AFP with any specific phylogenetic group. Viruses that were genetically identical or very similar to those associated with AFP in this study were isolated in different regions of Russia and were associated with acute respiratory diseases, enteroviral vesicular pharyngitis, HFMD and rarely aseptic virus meningitis.

## 4. Discussion

The main goal of AFP surveillance is the investigation of cases of a clinical syndrome that can be caused by multiple factors with the aim of identifying those cases that are associated with poliovirus—wild-type or derived from Sabin oral polio vaccine (OPV) strains (vaccine-related or vaccine-like). At the same time, the methodology of AFP surveillance and subsequent laboratory investigation allows the detection of NPEV in materials from AFP cases. NPEVs are ubiquitous and usually do not cause overt disease. Thus, the frequency of NPEV isolation from stool samples allows the assessment of the quality of surveillance for poliomyelitis and AFP. The frequency fluctuates in different countries depending on climatic and socio-economic conditions [14,15,16,17]. According to our data, in the Russian Federation, the proportion of AFP cases positive for NPEV was 3.2% in the period 2000–2019 (238 out of 7414). NPEV isolated from a non-sterile material (fecal sample, oropharyngeal swabs) may be an accidental finding that reflects the natural circulation of NPEV in the population and does not prove its etiological role in AFP. The link between an NPEV and AFP is well established for the types EV-A71 and EV-D68 [19]. The possibility of a large outbreak of paralytic disease, clinically indistinguishable from poliomyelitis, with fatal outcomes, but caused by non-polio EV-A71, was reported as early as 1975 by M.P. Chumakov and colleagues [44]. The capacity of this virus to cause AFP has been further confirmed [11]. EV-D68 has established itself as a human pathogen causing seasonal outbreaks with a large number of neurological manifestations in the form of acute flaccid myelitis in the United States and Europe since 2014 [45,46,47], necessitating the systematic monitoring of its circulation in some countries [48,49,50]. The etiological relationship between other NPEV types and AFP has not been definitively established. Despite the wide global circulation of the CVA2 virus, it is not commonly isolated from AFP cases, and available publications do not allow the determination of the role of CVA2 in the development of this syndrome. Over a 20-year period of AFP surveillance in the Russian Federation, we found only five AFP cases yielding CVA2. In three of five children, the disease resulted in the development of persistent residual paralysis, and the complex of clinical and laboratory data corresponded to poliomyelitis developing during poliovirus infection. It should be emphasized that all children were repeatedly (4 to 5 doses) immunized against poliomyelitis and did not have immunodeficiency disorders. No poliovirus or other enterovirus was isolated, and patients did not receive OPV recently (the last OPV dose being given at least two months prior to AFP onset). This allows the association of the AFP in these three patients with the CVA2 virus with a significant degree of confidence. The isolation of CVA in two other patients with clinical manifestations not compatible with poliomyelitis is a mundane finding that is not related in any way to the hypothetical ability of CVA2 to cause poliomyelitis. Indeed, if up to 20% of healthy children excrete NPEV [51,52]), then a comparable fraction of children with unrelated neurological disorders will be positive as well.

NPEVs can cause a wide range of symptoms, and a very similar disease can be caused by diverse types [7]. On the other hand, there have been descriptions of types (most prominently, EV-A71) or even distinct subtypes or phylogenetic groups causing specific clinical manifestations or having variable epidemiological properties [53,54,55]. Phylogenetic analysis of CV-A2 isolated from AFP cases did not reveal an association with a specific phylogenetic group. Viruses from AFP patients from this and other studies around the world were scattered across the phylogenetic tree. Thus, the severity and clinical manifestations of the disease are most likely determined by the individual characteristics of the infected child rather than by virus genetics. In this regard, it is worth noting that, in two children with persistent residual paralysis (N.T. and K.O.), AFP was preceded by other diseases (Table 1).

Tracing rare manifestations of common infections is generally a challenging task. The AFP surveillance system, which includes epidemiological and laboratory (virological) components, is well established, with a more than 30-year history of the Global Polio Eradication Initiative implementation. This system has clear criteria for detecting AFP cases, sampling and delivering materials for laboratory research and an algorithm for studying these samples [56]. Based on the goals of the poliomyelitis eradication program, the main objective of the surveillance is to detect poliovirus, and the main test materials are fecal samples [18]. However, owing to large sampling volumes and standardized methodology, there have been many scientific by-products of AFP surveillance, such as data on NPEV [14,17] and adenovirus circulation [57,58,59,60]. The future of AFP surveillance is currently debated, and some favor a more simple and versatile environmental surveillance program to trace both poliovirus and NPEV circulation. We have suggested previously that sewage surveillance may lack the sensitivity needed to ensure early poliovirus detection by orders of magnitude [55] and cannot fully substitute for AFP surveillance. This study exemplifies the additional value of AFP surveillance for the investigation of AFP etiology and studies on enterovirus pathogenicity. The value of AFP surveillance for AFP etiology studies could be further expanded by the systematic collection of additional material (oropharyngeal swabs, CSF) and use of molecular methods (PCR and high-throughput sequencing) for virus detection and identification. With the expected global certification of polio eradication [61], the elongation of AFP surveillance in the countries that conduct it will thus be reasonable.

## Figures and Tables

**Table 1:** Descriptive characteristics of confirmed AFP cases in patients yielding CVA2, Russian Federation, in 2001–2020.

Characteristic		Patients					
		H.A.	K.G.	D.A.	N.T.	K.O.	
Place of residence		Bryansk	Saratov	St. Petersburg	Vladimir region	Nizhny Novgorod region	
Gender		F	M	M	F	M	
Age, years		1	2	4	3	3	
Number of doses and the type of polio vaccine		2 OPV	5 OPV	4 IPV	3 IPV, 2 OPV	2 IPV, 3 OPV	
Date of the paralysis onset		5 October 2008	9 September 2008	23 September 2015	10 September 2019	23 September 2019	
Time from the last vaccination to the disease onset, months		13	5	24	8	2	
Time from the disease onset to the full manifestation of paralysis, days		10	4	1	1	2	
Body temperature at the disease onset		normal	39.0 °C	elevated, value unknown	39.0 °C	37.8 °C	
Localization of paralysis		left leg, right leg	left leg > right leg	left leg	left hand > right hand	quadriparesis (lower limbs > upper limbs)	
Proximal/distal		proximal	proximal > dystal	both	proximal > distal	both	
Other neurological signs			encopresis, enuresis				
Virus source and type isolated		feces, CVA2	feces, CVA2	feces, CVA2	feces, CVA2 oropharyngeal swab, CVA2	feces, CVA2	
CSF study results	Day since disease onset		unknown		09.12.19	27 September 2019	4 October 2019
	Cytosis, cells/mm^3^	no data	5	no data	3	61	12
	Protein, g/L		0.99		0.84	0.14	0.57
	Glucose, mmol/L		no data		no data	1.81	2.0
	Lymphocytes, %					80	84
	Neutrophils, %					20	16
Health condition before AFP		healthy	infectious mononucleosis one month prior to paresis	healthy	convalescence of bilateral focal pneumonia	aplasia of the left kidney, minor anomaly of heart development.	
Residual paralysis after 60 days from the onset		no	no	yes	yes	yes	
Clinical diagnosis at discharge *		Paraparesis of the lower limbs associated with CVA2. Organic lesion of central nervous system.	Polyradiculo-neuritis associated with non-polio enterovirus	Monoparesis of left lower limb	Enterovirus infection, cervical myelitis, upper flaccid paraparesis	Acute meningomyelitis	

* As provided in the medical records, not updated according to this paper’s results. IPV—inactivated poliovirus vaccine; OPV—oral poliovirus vaccine; CSF—cerebrospinal fluid.

## Data Availability

Not applicable.
